# A randomized triple-blind controlled clinical trial evaluation of sitagliptin in the treatment of patients with non-alcoholic fatty liver diseases without diabetes

**DOI:** 10.3389/fmed.2022.937554

**Published:** 2022-07-28

**Authors:** Azam Doustmohammadian, Ahmad Nezhadisalami, Fahimeh Safarnezhad Tameshke, Nima Motamed, Mansooreh Maadi, Mohammad Farahmand, Masoudreza Sohrabi, Cain C. T. Clark, Hossein Ajdarkosh, Amir Hossein Faraji, Mehdi Nikkhah, Elham Sobhrakhshankhah, Ramin Ebrahimi, Farhad Zamani

**Affiliations:** ^1^Gastrointestinal and Liver Diseases Research Center, Iran University of Medical Sciences, Tehran, Iran; ^2^Alimentary Tract Research Center, Clinical Sciences Research Institute, Ahvaz Jundishapur University of Medical Sciences, Ahvaz, Iran; ^3^Department of Social Medicine, Zanjan University of Medical Sciences, Zanjan, Iran; ^4^Department of Virology, School of Public Health, Tehran University of Medical Sciences, Tehran, Iran; ^5^Centre for Intelligent Healthcare, Coventry University, Coventry, United Kingdom; ^6^Department of Radiology, Firoozgar Hospital, Iran University of Medical Sciences, Tehran, Iran

**Keywords:** sitagliptin, NAFLD, clinical trial design, fibrosis scores, liver enzymes

## Abstract

The current study aimed to evaluate the efficacy of sitagliptin vs. placebo in treating non-alcoholic fatty liver disease (NAFLD). In a triple-blind randomized clinical trial, we assigned 120 eligible subjects with NAFLD to receive daily dosing of 50 mg sitagliptin (*n* = 60) or the placebo (*n* = 60) for 56 weeks and lifestyle modification in both groups. Laboratory and anthropometric outcomes were measured, and liver stiffness was assessed using a fibroscan. The primary outcome measures were changes from baseline in fibrosis scores and liver transferases. Out of 120 patients randomized into sitagliptin and placebo groups, 76 patients completed the trial, of whom 44 were in the sitagliptin and 32 in the placebo groups. Patients receiving sitagliptin showed a significant decrease in the fibrosis scores (*P* = 0.001). The reductions in the alanine aminotransferase (AST) (*P* = 0.036) and aspartate AST (*P* < 0.001) levels were also statistically significant. The effect of sitagliptin in reducing fibrosis scores was significantly greater in normal-weight and overweight individuals than in obese individuals (*p* = 0.036, and *p* = 0.018, respectively), whereas the effects of sitagliptin on AST levels were greater among overweight/obese patients (*p* = 0.028, and *p* = 0.016, respectively). Sitagliptin reduced fibrosis scores and liver enzymes in NAFLD patients after 56 weeks of therapy. The changes in fibrosis scores were more prominent in patients with normal weight and overweight than obese patients, whereas the effects on AST levels were greater among overweight/obese patients. Other randomized trials with larger sample sizes and longer treatment durations may be required before precise results can be reached.

**Clinical Trial Registration:**

[https://www.irct.ir/trial/46140], identifier [IRCT20140430017505N2].

## Introduction

The most common type of liver disease globally is a non-alcoholic fatty liver disease (NAFLD) (1), defined as hepatic steatosis without other causes of lipid accumulation in hepatocytes (2, 3). A meta-analysis in 2016 reported that the prevalence of NAFLD in adult populations in Europe, North America, and Asia was 23.7, 24.1, and 27.4%, respectively (4). According to research published in 2018, the prevalence of NAFLD in the adult population of Iran ranged from 20 to 40% (5).

NAFLD includes several stages, such as fatty steatosis to steatohepatitis, developing into liver fibrosis, and cirrhosis. NAFLD has been linked to metabolic syndrome, as the most well-known risk factor and several other metabolic diseases, including obesity, dyslipidemia, type 2 diabetes, and hypertension (6). NAFLD has recently been renamed metabolic-associated fatty liver disease (MAFLD) by an international expert consensus (7–9). Indeed, because insulin resistance (IR) and NAFLD are linked, diabetic people have a 4.7-fold higher prevalence of NAFLD than non-diabetic patients (10).

There is no definitive treatment for NAFLD, and no specific drug has been offered to treat it. The only therapeutic strategies are lifestyle modifications, including diet and physical activity/exercise, pointing to a weight loss of 5–7% (11, 12); although, IR modulation and various pharmacological approaches using existing drugs, including anti-obesity, anti-diabetic, antioxidants, and gastric cytoprotective agents, have been recently proposed as possible therapies in NAFLD and non-alcoholic steatohepatitis (NASH) (13, 14).

In the pathophysiology of NAFLD, metabolic abnormalities and insulin resistance play important roles (15, 16), such that several anti-diabetic therapies have been investigated in the treatment of NAFLD, with different results, including metformin (17, 18), tofogliflozin (19), empagliflozin (20), and liraglutide (21).

Dipeptidyl peptidase 4 (DDP-4) is one of the key molecules implicated in the pathogenesis of chronic liver illnesses, like NAFLD, even before diabetes develops (22). Sitagliptin is a dipeptidyl peptidase-4 (DPP-4) inhibitor, increasing glucagon-like peptide-1 (GLP-1) levels and inhibiting glucagon release, enhancing insulin secretion, and ameliorating liver enzymes (23–26).

Clinical research studies have confirmed that sitagliptin significantly improves glycemic parameters, lipid profiles, and liver function of diabetic patients with NAFLD (27–29), although other studies reported contradictory results (30, 31).

In a study of 30 NAFLD patients, Iwasaki et al. noted that 4 months of treatment with 50 mg/day of sitagliptin significantly decreased liver transferases and improved diabetes parameters (27). In an open-label, single-arm, observational pilot study that included 15 patients, results showed that treatment for 1 year with sitagliptin was associated with a significant reduction in NASH scores and hepatic steatosis improvement (29).

However, there is limited evidence of the influence of sitagliptin on hepatic inflammation and fibrosis (30, 32, 33), and only one study has been done on patients without diabetes (34). Relevant randomized control trials and long-term studies are still needed to verify the efficacy and safety of sitagliptin (35). Therefore, the current work aimed to examine the effectiveness of sitagliptin in treating NAFLD compared to placebo in patients without diabetes. The inclusion of this group of NAFLD patients in our investigation was based on the assumption that, in the absence of simultaneous diabetes, several metabolic and other confounding factors could influence the response of the fatty liver disease to any recommended treatment.

## Materials and methods

### Study design, setting, and population

A randomized, triple-blind clinical trial was conducted in two tertiary referral teaching medical centers in Amol and Tehran, Iran. Adults diagnosed with NAFLD were enrolled in this study from January 2019 to May 2020. Inclusion criteria were patients aged ≥ 18 years old whose primary ultrasound was scored as grades 2 and 3 and whose liver enzyme levels based on ALT were above 20 in women and above 30 in men.

Any other cause of liver diseases, such as viral hepatitis, autoimmune hepatitis, hemochromatosis, Wilson disease, liver cirrhosis, and drug-induced liver injury, was ruled out. Patients would be excluded from the trial if they had one of the following conditions: Individuals on a specific dietary or physical activity regimen (due to a specific disease, weight loss, or professional exercise), taking Vitamin E supplement, history of excessive alcohol consumption (> 10 grams per day), history of taking drugs that cause hepatic steatosis (e.g., amiodarone, methotrexate, tamoxifen, glucocorticoids, valproate, anti-retroviral agents for HIV), diagnosed diabetes, known case of malignancy, non-cooperative patients, pregnant women, and breastfeeding mothers. All of the patients provided written informed consent prior to study participation.

### Randomization and allocation concealment

In order to exclude confounding factors, randomization was stratified by age, sex, and ultrasound grade. Patients were divided into 12 groups, including subjects with grades 2 and 3 in three age categories: under 40 years, 40–59 years, and ≥ 60 years in both genders, separately. Then, randomization codes were assigned for each group, and patients were randomly allocated to one of the two treatment arms, including case (sitagliptin + lifestyle modification) and control (placebo + lifestyle modification) groups. Patients, researchers, and analyzers were blinded to allocation.

### Primary and secondary outcome measurements

The primary outcome measures included hepatic fibrosis and liver enzymes, such as alanine aminotransferase (ALT), aspartate aminotransferase (AST), gamma-glutamyl transferase (GGT), and alkaline phosphatase (ALP), were assessed using changes from baseline to week 56. Several secondary outcome measures included FBS, HOMA-IR, Insulin, and serum lipid profiles, including total cholesterol, low-density lipoprotein (LDL) cholesterol, high-density lipoprotein (HDL) cholesterol, and triglyceride.

### Non-alcoholic fatty liver disease diagnosis

For hepatic steatosis, ultrasonography was utilized as a first-line investigation, providing a qualitative assessment of fatty infiltration of the liver. Patients were scanned in longitudinal, transverse, and oblique scanning planes while lying down and in the left posterior oblique posture. Fatty liver on ultrasonography was defined as normal, mild, and moderate to severe. This classification was based on the echogenicity of the liver during ultrasonography, liver-to-kidney contrast, and bright gallbladder and vessel walls definition (36).

Fibroscan evaluation was applied for all participants who had fatty liver confirmed by ultrasonography. Fibroscan assessment is considered a preferred non-invasive method to apply liver stiffness measurement (LSM) and steatosis level by control attenuated parameter (CAP). According to the standard protocol, two trained specialists, blinded to the treatment group in each research center, performed the fibro scan using a Fibroscan device (Fibro Scan; Supersonic Axiplore Ultimate Paris, France) (37).

Patients were considered to have NAFLD based on a fibroscan if the fibrosis score was more than 6 kpa with absent-mild, moderate, and severe fibrosis, defined as a fibrosis score lower than 6 kpa (F0-F1), between 6 and 9.1 kpa (F2), and more than 9.1 kpa (F3), respectively (38, 39).

### Laboratory and anthropometric measurements

The following procedures were used to assess laboratory outcomes at baseline and after 56 weeks of intervention. At baseline and the end of the study, 5 ml of blood was drawn from all patients to measure serum insulin levels, fasting plasma glucose (FPG), lipid profiles, and liver biochemical tests (pars biochemical kits using the photometric method). HOMA-IR formula was calculated as [fasting plasma glucose (mg/dl) serum insulin level (mU/L)]/405 (40). Weights and heights were measured at baseline, and body mass index (BMI) was computed and classified according to the World Health Organization (WHO) classification into four groups: Less than 18.5 as underweight, 18.5–25 as normal, 25.0–30 as overweight, and 30.0 kg.m^2^ or over as obese (41).

### Intervention protocol

Patients were randomly divided into case (sitagliptin + lifestyle modification) and control (placebo + lifestyle modification) groups. A computer-based technique was employed to use block randomization, with a block of 4, and subjects were randomized 1:1 to receive sitagliptin or placebo.

During the intervention period, patients in the sitagliptin group were given 50 mg of sitagliptin (Ziptin, Abidi, Iran), whereas those in the placebo group were given 1 mg of folic acid once daily. A trained person, who was blind to the medication and patients, distributed medication packages in the same shape packaging, without labeling, to participants. A total of 120 patients were randomly assigned to sitagliptin (*n* = 60) and placebo (*n* = 60) groups for 56 weeks, along with advising for lifestyle modifications for both groups (42).

At the end of the trial, patients’ adherence to their medicine use was examined using the pill counting method. Compliance was deemed appropriate if the leftover medications were less than 20%. Patients were contacted regularly and screened for side effects throughout and after the treatment to enhance adherence to the medication and follow-up examinations. The only reported side effect among patients in the sitagliptin arm was headache (*n* = 1).

Various patients’ information, such as demographic, anthropometric, and laboratory data, was obtained and recorded by a trained person who was blind to grouping. All individuals’ laboratory data were gathered at baseline and after 56 weeks of therapy. Finally, the two groups’ laboratory and fibrosis results were compared before and after the intervention.

### Ethical issue

The Helsinki Declaration’s guidelines were followed in the design of this study. The Medical Ethics Committee of the Iran University of Medical Science reviewed, approved, and supervised this work (reference number IR.IUMS.REC.1397.1062). The Iranian Registry of Clinical Trials also authorized and registered this protocol (IRCT20140430017505N2, https://www.irct.ir/trial/46140). Written informed consent was obtained from participants before study commencement.

### Sample size calculation and statistical analysis

According to an effect size (Cohen’s D) equal to 0.5 (based on 4 kpa changes in ultrasound grade), with an α of 0.05 and power of 80%, the required sample size was 60 per group (total of 120 participants) (43, 44). Continuous and categorical variables were presented as median (IQR) and n (%), respectively. The Wilcoxon rank-sum test, χ^2^-test, or Fisher’s exact test was used to compare differences between two independent groups. Furthermore, the Wilcoxon signed-rank test was used to compare differences between before and after interventions. A two-sided α of less than 0⋅05 was considered statistically significant. Statistical analyses were conducted using R statistical software, version 4.1.1 (2021-08-10).

## Results

### Study flow

**FIGURE 1:**
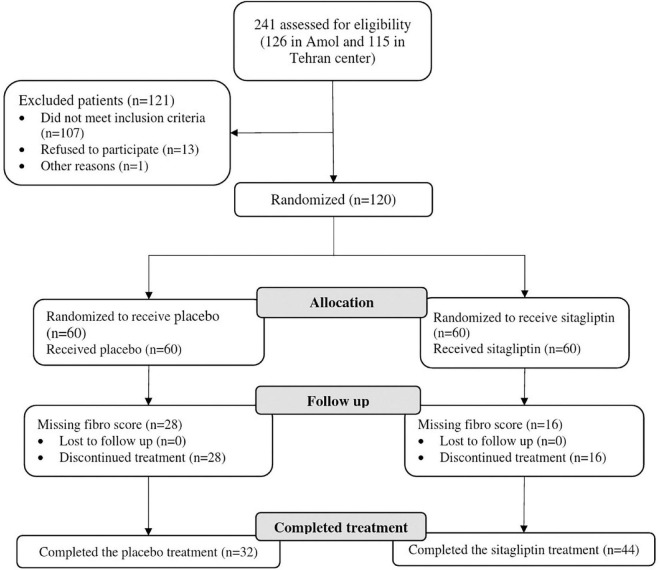
Participants’ CONSORT flowchart.

A total of 241 patients (126 in Amol and 115 in Tehran) were assessed for eligibility. One hundred twenty subjects were finally included in the study and were monitored from January 2019 to May 2020. Out of 120 patients randomized into sitagliptin (*n* = 60) and placebo groups (*n* = 60), 76 patients from both centers completed the trial, of whom 44 were in the sitagliptin and 32 in the placebo groups. Figure 1 depicts the trial patient’s assignment and flow chart based on the CONSORT flowchart (45). Dropouts from the placebo arm were not linked to any adverse events in the trial.

### Baseline characteristics

The median (IQR) ages of sitagliptin and placebo groups were 45.0 (36.8, 53.2) and 39.0 (33.8, 44.2) years, respectively (*P* = 0.03). There were 6 (13.6%) normal weight, 18 (40.9%) overweight, and 20 (45.5%) obese patients in the sitagliptin group. In terms of demographic features and laboratory data, there was no statistically significant difference between the two groups, except for age, which was higher in the sitagliptin group than in the placebo group. The baseline demographic and laboratory characteristics of participants are shown in Table 1. Considering the sex dysmorphism of NAFLD (male predominance), a separate analysis for sex was also conducted and reported in Supplementary Tables 1–3. Demographic features, and clinical and laboratory characteristics of the study groups by sex at baseline are presented in Supplementary Table 1; females in the sitagliptin group were older than the placebo group (*P* = 0.003), and males in the sitagliptin group had higher fibrosis scores than the placebo group (*P* = 0.018), without significant differences for other variables.

**TABLE 1:** Baseline demographic and laboratory characteristics of participants.^a^

Variables		Sitagliptin group (*n* = 44)	Placebo group (*n* = 32)	*P*-value^b^
**Demographic data**				
**Sex; n (%)**				0.99
	Male	33 (75)	24 (75)	
	Female	11 (25)	8 (25)	
**Age (years)**		45.0 (36.8, 53.2)	39.0 (33.8, 44.2)	0.03
**Height**		168.0 (163.0, 177.2)	170.5 (162.0, 179.2)	0.59
**Weight**		84.0 (76.8, 92.0)	82.5 (74.5, 94.1)	0.65
**BMI (kg/m2)**		29.6 (26.5, 32.7)	28.8 (26.4, 31.4)	0.67
**Metabolic factors**				
BMI categories; n (%)				0.46
	Normal	6 (13.6)	3 (9.4)	
	Overweight	18 (40.9)	18 (56.2)	
	Obese	20 (45.5)	11 (34.4)	
FBS (mg/dl)		101.5 (96.8, 114.0)	107.0 (98.0, 112.2)	0.97
Insulin (mU/L)		13.3 (9.6, 18.4)	14.4 (10.7, 17.8)	0.48
HOMA-IR		3.5 (2.4, 4.7)	3.8 (2.6, 4.6)	0.44
**Serum lipid levels**				
Total cholesterol (mg/dl)		189.0 (164.8, 221.8)	201.0 (174.2, 238.2)	0.60
LDL-cholesterol (mg/dl)		121.0 (110.0, 137.2)	125.0 (111.5, 137.0)	0.86
HDL-cholesterol (mg/dl)		40.5 (33.8, 45.2)	42.0 (37.0, 44.0)	0.45
Triglyceride (mg/dl)		145.5 (109.8, 201.2)	149.0 (106.0, 222.8)	0.88
**Serum biochemical levels**				
ALT (IU/L)		44.0 (34.5, 70.0)	38.0 (33.0, 56.2)	0.34
AST (IU/L)		35.5 (28.0, 49.2)	31.0 (26.8, 41.2)	0.14
GGT (IU/L)		43.5 (36.5, 55.2)	40.5 (24.0, 51.2)	0.06
ALKP (IU/L)		201.0 (157.5, 230.5)	175.5 (159.8, 214.2)	0.37
Ferritin (μg/l)		108.5 (37.5, 172.0)	95.0 (47.8, 217.2)	0.92
**Liver histology**				
Ultrasound; n (%)				0.77
	Grade 1	10 (23)	9 (28)	
	Grade 2	14 (32)	8 (25)	
	Grade 3	20 (45)	15 (47)	
Fibrosis score (kPa)		6.2 (6.0, 7.3)	6.0 (5.0, 6.9)	0.045

^a^Median (IQR); n (%).

^b^Wilcoxon rank-sum test; Pearson’s Chi-squared test; Fisher’s exact test.

BMI, body mass index; FBS, fasting blood sugar; HOMA-IR, homeostasis model of insulin resistance; LDL, low-density lipoprotein; HDL, high-density lipoprotein; ALT, alanine aminotransferase; AST, aspartate aminotransferase; GGT, gamma-glutamyltransferase. Statistically significant results are reported in bold.

### Primary outcomes

#### Changes in hepatic fibrosis and liver enzymes

The decrease of the median fibrosis scores in patients receiving sitagliptin was statistically significant compared to individuals receiving placebo. The median (IQR) of fibrosis scores, before and after intervention with sitagliptin, were 6.25 (5.97, 7.30) and 6.00 (5.40, 6.85), respectively. The decrease of the median (IQR) fibrosis scores in patients receiving sitagliptin was statistically significant [median before 6.25 (5.97, 7.30) vs. median after 6.00 (5.40, 6.85), *P* = 0.001)], but not in individuals receiving placebo (*P* = 0.19).

Patients receiving sitagliptin showed a significant decrease in ALT (*P* = 0.036) and AST level (*P* < 0.001). While in the placebo group, only AST decreased significantly (*p* = 0.019). However, the reduction in AST level was 2.4 times greater in sitagliptin-treated patients than in placebo-treated individuals, which was clinically significant. Table 2 shows the changes in laboratory data in both groups before and after treatment.

**TABLE 2:** Changes in fibrosis score and laboratory data in the sitagliptin group (*n* = 44) and the placebo group (*n* = 32) before and after the intervention.^a^

Variables	Groups	Before intervention median (IQR)	After intervention median (IQR)	*P*-value^b^
Insulin (Mu/L)	Sitagliptin	13 (10, 18)	11 (9, 16)	0.11
	Placebo	14 (11, 18)	13 (11, 17)	0.54
HOMA-IR	Sitagliptin	3.53 (2.40, 4.70)	2.93 (2.14, 4.29)	0.09
	Placebo	3.83 (2.64, 4.57)	3.34 (2.67, 4.44)	0.56
FBS (mg/dl)	Sitagliptin	102 (97, 114)	101 (97, 108)	0.63
	Placebo	107 (98, 112)	101 (94, 108)	0.92
Total cholesterol (mg/dl)	Sitagliptin	189 (165, 222)	176 (158, 205)	0.12
	Placebo	201 (174, 238)	174 (156, 204)	0.030
LDL-cholesterol (mg/dl)	Sitagliptin	121 (110, 137)	106 (92, 131)	0.023
	Placebo	125 (112, 137)	105 (98, 135)	0.19
HDL-cholesterol (mg/dl)	Sitagliptin	40 (34, 45)	40 (40, 42)	0.72
	Placebo	42 (37.0, 44.0)	42 (40.0, 44.5)	0.52
Triglyceride (mg/dl)	Sitagliptin	146 (110, 201)	136 (118, 202)	0.18
	Placebo	149 (106, 223)	134 (102, 183)	0.69
ALT (IU/L)	Sitagliptin	44 (34, 70)	40 (24, 52)	0.036
	Placebo	38 (33, 56)	35 (30, 50)	0.21
AST (IU/L)	Sitagliptin	36 (28, 49)	24 (19, 36)	< 0.001
	Placebo	31 (27, 41)	26 (20, 32)	0.019
GGT (IU/L)	Sitagliptin	44 (36, 55)	42 (40, 48)	0.43
	Placebo	40 (24, 51)	41 (35, 49)	0.90
ALKP (IU/L)	Sitagliptin	201 (158, 230)	192 (156, 231)	0.60
	Placebo	176 (160, 214)	179 (168, 198)	0.25
Ferritin (μg/l)	Sitagliptin	108 (38, 172)	105 (67, 170)	0.081
	Placebo	95 (48, 217)	66 (40, 108)	0.22
Fibrosis score, Med (IQR)	Sitagliptin	6.25 (5.97, 7.30)	6.00 (5.40, 6.85)	0.001
	Placebo	6.00 (5.00, 6.93)	5.45 (4.80, 6.30)	0.19

^a^Median (IQR).

^b^Wilcoxon signed rank test with continuity correction; Wilcoxon signed rank exact test.

FBS, fasting blood sugar; HOMA-IR, homeostasis model of insulin resistance; LDL, low-density lipoprotein; HDL, high-density lipoprotein; ALT, alanine aminotransferase; AST, aspartate aminotransferase; GGT, gamma-glutamyltransferase.

In male participants, there was a significant decrease in the level of ALT, AST, and fibrosis scores after treatment. There were no significant differences among females (see Supplementary Table 3).

#### Changes in hepatic fibrosis and liver enzymes by body mass index classification

Changes in fibrosis scores and liver enzyme levels according to the BMI classification in sitagliptin and placebo-treated patients are shown in Table 3. The effect of sitagliptin in reducing fibro scores was significantly greater in normal-weight and overweight individuals than in obese individuals (*p* = 0.036, and *p* = 0.018, respectively), whereas the effects on AST levels were higher among overweight/obese patients (*p* = 0.028, and *p* = 0.016, respectively). However, separate analysis for sex showed that the effect of sitagliptin in reducing fibro scores (*p* = 0.032) and AST level (*p* = 0.039 and *p* = 0.009, respectively) was significantly greater among overweight and obese males. The differences in these variables were not significant in females (Supplementary Table 3).

**TABLE 3:** Changes in outcome in the treatment group (*n* = 44) and placebo group (*n* = 32) based on the BMI status.^a^

Variables	Groups	BMI status	Before intervention, median (IQR)	After intervention, median (IQR)	*P*-value^b^
ALT (IU/L)	Sitagliptin	Normal	63 (40, 74)	41 (26, 52)	0.59
		Overweight	42 (34, 54)	40 (24, 50)	0.16
		Obese	47 (35, 71)	38 (24, 55)	0.26
	Placebo	Normal	56 (50, 61)	42 (38, 46)	0.25
		Overweight	40 (34, 68)	34 (30, 50)	0.48
		Obese	33 (30, 46)	32 (22, 53)	0.45
AST (IU/L)	Sitagliptin	Normal	43 (28, 61)	31 (21, 51)	0.58
		Overweight	32 (28, 44)	24 (20, 37)	0.028
		Obese	39 (29, 44)	24 (17, 33)	0.016
	Placebo	Normal	42 (37.5, 43.0)	29 (28.0, 36.5)	0.50
		Overweight	30 (28, 47)	26 (20, 31)	0.055
		Obese	27 (26, 34)	22 (17, 28)	0.38
GGT (IU/L)	Sitagliptin	Normal	62 (57, 66)	51 (42, 64)	0.62
		Overweight	41 (35, 52)	41 (37, 44)	0.66
		Obese	42 (36, 51)	42 (40, 46)	0.85
	Placebo	Normal	46 (43.50, 48.50)	48 (45.00, 49.00)	> 0.99
		Overweight	35 (20, 49)	38 (29, 43)	> 0.99
		Obese	41 (30, 52)	41 (40, 47)	> 0.99
ALKP (IU/L)	Sitagliptin	Normal	207 (164, 270)	164 (146, 195)	0.062
		Overweight	198 (155, 205)	175 (159, 208)	0.42
		Obese	212 (156, 228)	213 (155, 256)	0.17
	Placebo	Normal	176 (139, 180)	195 (151, 196)	0.25
		Overweight	170 (160, 211)	179 (168, 198)	0.35
		Obese	184 (158, 244)	177 (175, 205)	> 0.99
Fibro score	Sitagliptin	Normal	7.05 (6.05, 8.72)	5.45 (5.18, 7.75)	0.036
		Overweight	6.15 (5.82, 7.15)	5.55 (5.00, 6.27)	0.018
		Obese	6.45 (6.00, 7.08)	6.00 (5.70, 6.85)	0.24
	Placebo	Normal	4.60 (4.60, 6.30)	6.60 (5.30, 6.70)	> 0.99
		Overweight	5.50 (5.00, 6.07)	5.25 (4.73, 6.00)	0.67
		Obese	6.90 (6.20, 7.10)	5.50 (5.00, 7.00)	0.10

^a^Median (IQR).

^b^Wilcoxon signed rank test with continuity correction; Wilcoxon signed rank exact test.

### Secondary outcomes: Changes in ferritin, lipid profile, and glucose parameters

LDL-cholesterol was significantly decreased in patients receiving sitagliptin compared to the placebo group (*P* = 0.023), while the effect of sitagliptin on the reduction of the other secondary outcomes was not statistically significant. A significant increase in serum ferritin level was observed in the sitagliptin group among females, after the intervention (*P* = 0.01). There were no significant differences for other variables (Supplementary Table 2).

## Discussion

To our knowledge, this is the first multicenter randomized, triple-blind, placebo-controlled clinical trial to evaluate the effect of sitagliptin in NAFLD treatment. Our findings showed that sitagliptin, combined with lifestyle modification, reduced fibrosis score, ALT, and AST levels, over 56 weeks of treatment.

Existing evidence evaluating the effects of sitagliptin on fibrosis score and liver transferases in NAFLD is equivocal. Nevertheless, consistent with our results, Alam et al. (34), in an open-label randomized control trial on 40 NASH patients diagnosed with dual-pass liver biopsy, found that intervention with sitagliptin, daily for 1 year, combined with lifestyle modification, improved hepatic histological and fibrosis of non-alcoholic steatosis patients, as compared with lifestyle modification only. Our results also agreed with Sayari et al. (46), who reported that treatment with sitagliptin in NAFLD patients for 16 weeks significantly reduced ALT and AST levels.

The results of several small-scale, placebo-controlled RCTs conducted in patients with NAFLD indicated that 24 weeks of sitagliptin therapy might not have a beneficial effect on liver fibrosis and transferases (21, 30, 47). Indeed, it has been posited that long-term treatment periods of 1 year or more may be needed to observe such effects (34).

Empirical evidence suggests that sitagliptin might improve steatosis by suppressing lipogenic and gluconeogenic pathways through the inhibitation of DPP-4 and increasing levels of biological activity of GLP-1 and GIP (48, 49). Indeed, researchers have concluded that sitagliptin may have more robust efficacy than weight loss in improving non-alcoholic steatohepatitis, irrespective of diabetes (34).

In the current study, the fibrosis changes were significantly more prominent in patients with normal weight and overweight at baseline, whereas the effects of sitagliptin on AST level were greater among overweight/obese patients. To the best of our knowledge, the role of baseline BMI as a predictor of NAFLD patients’ response to sitagliptin has not been previously reported. Our results showed that lower baseline BMI may lead to better fibrosis scores in patients receiving sitagliptin. This pattern was reversed in the effect of sitagliptin on the AST level. In fact, the extant literature suggests that lifestyle modifications have a greater impact on downgrading fibroscan values and ALT than other NAFLD biomarkers (50).

Obesity may attenuate the liver fibrosis-lowering effect of sitagliptin in NAFLD patients. In some studies (51, 52), GLP-1 levels in response to increased carbohydrate intake in obese patients have been reduced, leading to sitagliptin exerting a less potent effect on the decrease in fibrosis score with increasing BMI. In addition, obese patients may not adhere to their lifestyle modification as well as normal-weight patients, although we have no objective data to support this.

Among the secondary outcome measures, sitagliptin significantly reduced LDL-cholesterol in the sitagliptin group and did not affect other lipid profiles, IR parameters, and ferritin. Concordant with our study, Derosa et al. reported that patients receiving sitagliptin, after 7 years of therapy, experienced a greater decrease in total cholesterol and LDL-cholesterol compared to baseline (53). In contrast, in other studies, sitagliptin elicited no significant change in serum level of LDL-cholesterol, despite a reduction in TG, total cholesterol, and HDL cholesterol (54).

A retrospective study by Horton et al. indicated that treatment with sitagliptin for 90–365 days substantially decreased serum levels of TG, total cholesterol, and LDL-cholesterol, except for HDL cholesterol, in individuals with type 2 diabetes (55). Overall, either alone or in combination, sitagliptin resulted in a better lipid profile. The favorable effects of sitagliptin on serum lipid profiles might contribute to its protective effects on gastric inhibitory polypeptide (GIP) and glucagon-like peptide-1 (GLP-1) in improving lipid metabolism. On the other hand, the other effects of sitagliptin on glycemic control and insulin resistance, weight reduction, or delayed stomach emptying can putatively exhibit beneficial effects on lipoprotein metabolism (56).

The strengths of this study include the randomized controlled design, which facilitates a low probability of selection bias and residual confounding. The implementation of a well-validated outcome assessment, such as a fibroscan, is another strength of the study. Further, the present study was conducted through multicenter tertiary care, thus favorably supporting the generalizability of our work. However, there are limitations to the present study that should be considered. The major limitation of this study is the relatively small sample size recruited and the high dropout rate. Indeed, the second 6 months of the trial coincided with the COVID-19 pandemic, which had significant effects on the dropout of participants. This limitation reduces the power of the study to identify significant effects; however, even with the limited sample, significant improvements in several outcomes were observed. This suggests that the effect of the sitagliptin on these measures may be more potent than estimated in prior power calculations. The other main limitations of the study included the lack of assessment of abdominal obesity by WC or WHR; indeed, although visceral fat excess is the main mechanism of NAFLD, lifestyle modifications, including dietary intake and physical activity, there was no strict protocol in this study, and weight status was not evaluated at the end of the treatment. One of the potential limitations in all clinical trials is the contamination across groups. Contamination of control participants has two related effects. It reduces the point estimate of an intervention’s effectiveness, and this apparent reduction may lead to a type II error- rejection of an effective intervention as ineffective because the observed effect size was neither statistically nor clinically significant (57). However, this issue was mitigated by the triple-blind design in the current study. Further, the effect of social desirability due to the cultural context of our society was another limitation that could affect how the study participants responded to the question of alcohol consumption history. However, we tried to mitigate this bias effect by reassuring patients about the confidentiality of their information and active and ongoing communication with participants. Furthermore, shear-wave elastography was used to determine NAFLD severity rather than liver biopsy, which is the gold-standard procedure (58). Since liver biopsy is an invasive procedure carrying potential risks of several complications (59), newer commercially available equipment for liver elastography, such as shear-wave elastography, gives enhanced diagnostic accuracy compared to other elastographic techniques and minimizes different physical limits of the approach, such as the presence of obesity (60, 61). Finally, we used additional glycemic control markers, such as FBS, serum insulin, and HOMA-IR, instead of measuring HbA1C, which is an index for overall glycemia.

## Conclusion

Sitagliptin reduced fibrosis scores and liver enzymes in NAFLD patients after 56 weeks of therapy. The changes in fibrosis scores were more prominent in patients with normal weight and overweight than obese patients, whereas the effects on AST levels were greater among overweight/obese patients. Further randomized trials with larger sample sizes and longer treatment durations may be required before consensus can be reached.

## Data availability statement

The raw data supporting the conclusions of this article will be available from the corresponding author on reasonable request. Requests to access these data sets should be directed to FZ, zamani.farhad@gmail.com.

## Ethics statement

The studies involving human participants were reviewed and approved by the Iran University of Medical Sciences (IUMS) ethical committee (No. IR.IUMS.REC.1397.1062). The patients/participants provided their written informed consent to participate in this study.

## Author contributions

FZ, AN, NM, HA, MN, AF, ES, and RE were responsible for the study concept and design. FZ and AD had full access to all data and took responsibility for the integrity of the data and the accuracy of the data analysis. MM, FS, and MS involved in data collection. NM, CC, and MF analyzed and interpreted the data. AD and AN wrote the initial draft of the manuscript. FZ was the guarantor and takes responsibility for the manuscript as a whole. All authors revised the manuscript critically for important intellectual content and approved the final manuscript.
