# Supplementary material for: A randomized triple-blind controlled clinical trial evaluation of sitagliptin in the treatment of patients with non-alcoholic fatty liver diseases without diabetes
Source: Front Med (Lausanne). 2022 Jul 28;9:937554. doi: 10.3389/fmed.2022.937554 (PMC9365981; doi:10.3389/fmed.2022.937554)
Supplement: Supplementary file 1 [file Data_Sheet_1.DOCX]

**Supplementary Table 1.Baseline demographic and laboratory characteristics of participants by sex ^a^**

|  | **Male (n=57 )** | | | **Female (n=19)** | | |
| --- | --- | --- | --- | --- | --- | --- |
| **Variables** | **Sitagliptin group**  **(n = 33)** | **Placebo group**  **(n = 24)** | **P value ^b^** | **Sitagliptin group**  **(n = 11)** | **Placebo group**  **(n = 8)** | **P value ^b^** |
| **Demographic data** |  |  |  |  |  |  |
| **Age (years)** | 43.0 (35.0, 46.0) | 39.0 (33.8,44.2) | 0.32 | 57.0 (54.0, 58.5) | 39.0 (32.8, 44.5) | 0.003 |
| **Height** | 172.0 (167.0, 178.0) | 173.5 (169.8, 180.5) | 0.48 | 160.0 (158.0, 162.5) | 158.0 (157.8, 160.0) | 0.45 |
| **Weight** | 84.0 (78.0, 98.0) | 81.0 (74.9, 93.4) | 0.29 | 84.0 (67.5, 88.2) | 87.0 (74.1, 94.2) | 0.28 |
| **BMI (kg/m2)** | 29.0 (27.8, 31.9) | 27.3 (26.2, 29.9) | 0.23 | 32.8 (25.6, 33.8) | 33.0 (29.4, 36.3) | 0.49 |
| **Metabolic factors** |  |  |  |  |  |  |
| BMI categories; n (%) |  |  | 0.58 |  |  | 0.27 |
| Normal | 3 (9.1%) | 3 (12%) |  | 3 (27) | 0 (0) |  |
| Overweight | 17 (52%) | 15 (62%) |  | 1 (9.1) | 3 (38) |  |
| Obese | 13 (39%) | 6 (25%) |  | 7 (64) | 5 (62) |  |
| FBS (mg/dl) | 105.0 (99.0, 119.0) | 110.0 (101.5, 113.2) | 0.91 | 100.0 (95.0, 100.0) | 96.5 (89.5, 101.5) | 0.71 |
| Insulin (mU/L) | 13.3 (9.3, 17.9) | 13.7 (10.6, 15.9) | 0.75 | 12.9 (10.4, 18.4) | 16.2 (12.8, 20.8) | 0.41 |
| HOMA-IR | 3.7 (2.4, 4.7) | 3.8 (2.6, 4.4) | 0.67 | 3.2 (2.4, 3.9) | 3.9 (2.7, 5.2) | 0.41 |
| **Serum lipid levels** |  |  |  |  |  |  |
| Total cholesterol (mg/dl) | 187.0 (166.0, 224.0) | 210.0 (189.2, 247.5) | 0.18 | 198.0 (153.5, 215.5) | 168.5 (142.0, 187.2) | 0.30 |
| LDL-cholesterol (mg/dl) | 122.0 (110.0, 137.0) | 130.5 (118.0, 138.5) | 0.39 | 117.0 (108.0, 138.0) | 117.5 (104.0, 122.8) | 0.39 |
| HDL-cholesterol (mg/dl) | 38.0 (32.0, 44.0) | 40.5 (35.8, 44.5) | 0.36 | 42.0 (41.0, 47.0) | 42.5 (41.8, 43.2) | 0.97 |
| Triglyceride (mg/dl) | 151.0 (115.0, 213.0) | 165.5 (138.5, 242.5) | 0.52 | 123.0 (81.5, 163.0) | 96.0 (87.0, 112.2) | 0.44 |
| **Serum biochemical levels** |  |  |  |  |  |  |
| ALT(IU/L) | 46.0 (36.0, 74.0) | 43.5 (35.0, 58.5) | 0.34 | 36.0 (26.5, 56.0) | 29.0 (21.8, 37.5) | 0.30 |
| AST(IU/L) | 36.0 (29.0, 45.0) | 31.5 (28.0, 41.2) | 0.22 | 31.0 (26.5, 51.5) | 26.0 (22.8, 39.2) | 0.34 |
| GGT(IU/L) | 48.0 (35.0, 55.0) | 37.0 (23.5, 51.2) | 0.06 | 42.0 (38.5, 53.5) | 42.5 (36.0, 51.8) | 0.68 |
| ALKP(IU/L) | 195.0 (150.0, 216.0) | 179.5 (165.2, 221.5) | 0.97 | 225.0 (167.0, 254.0) | 163.5 (154.5, 185.5) | 0.11 |
| Ferritin (µg/l) | 132.0 (69.0, 182.0) | 118.5 (66.5, 228.0) | 0.95 | 52.0 (25.5, 88.0) | 30.0 (12.2, 85.0) | 0.44 |
| **Liver histology** |  |  |  |  |  |  |
| Ultrasound; n (%) |  |  | 0.97 |  |  | 0.33 |
| Grade 1 | 9 (27) | 6 (25 |  | 1 (9.1) | 3 (38) |  |
| Grade 2 | 10 (30) | 7 (29) |  | 4 (36) | 1 (12) |  |
| Grade 3 | 14 (42) | 11 (46) |  | 6 (55) | 4 (50) |  |
| Fibrosis score (kPa) | 6.2 (6.0, 7.3) | 5.6 (5.0, 6.4) | 0.018 | 6.8 (6.0, 7.2) | 6.7 (6.0, 7.2) | 0.87 |

^a^ Median (IQR); n (%), ^b^ Wilcoxon rank-sum test; Pearson's Chi-squared test; Fisher's exact test, Abbreviations: BMI, body mass index; FBS, fasting blood sugar; HOMA-IR: homeostasis model of insulin resistance; LDL: low-density lipoprotein; HDL: high-density lipoprotein; ALT: alanine aminotransferase; AST: aspartate aminotransferase; GGT: gamma-glutamyltransferase. Statistically significant results are reported in bold.

**Supplementary Table 2.Changes in fibrosis score and laboratory data in the sitagliptin group (n=44) and the placebo group (n=32) before and after the intervention by sex ^a^**

|  |  | **Male (n=57 )** | | | **Female (n=19)** | | |
| --- | --- | --- | --- | --- | --- | --- | --- |
| **Variables** | **Groups** | **Before intervention**  **Median (IQR)** | **After intervention**  **Median (IQR)** | **P-value ^b^** | **Before intervention**  **Median (IQR)** | **After intervention**  **Median (IQR)** | **P-value ^b^** |
| Insulin (Mu/L) | Sitagliptin | 13 (9, 18) | 11 (8, 17) | 0.21 | 12.9 (10.4, 18.4) | 11.0 (9.6, 14.2) | 0.29 |
|  | Placebo | 14 (11, 16) | 13 (10, 16) | 0.78 | 16.2 (12.8, 20.8) | 12.7 (11.7, 17.0) | 0.47 |
| HOMA-IR | Sitagliptin | 3.69 (2.42, 4.73) | 3.14 (1.95, 4.55) | 0.18 | 3.20 (2.38, 3.92) | 2.64 (2.43, 3.27) | 0.38 |
|  | Placebo | 3.83 (2.60, 4.40) | 3.34 (2.55, 4.63) | 0.82 | 3.89 (2.72, 5.19) | 3.07 (2.85, 3.93) | 0.58 |
| FBS (mg/dl) | Sitagliptin | 105 (99, 119) | 103 (98, 111) | 0.43 | 100 (95, 100) | 98 (93, 104) | 0.89 |
|  | Placebo | 110 (102, 113) | 104 (96, 112) | 0.78 | 96 (90, 102) | 94 (92, 104) | 0.83 |
| Total cholesterol (mg/dl) | Sitagliptin | 187 (166, 224) | 176 (162, 205) | 0.10 | 198 (154, 216) | 187 (146, 204) | 0.70 |
|  | Placebo | 210 (189, 248) | 180 (160, 206) | 0.036 | 168 (142, 187) | 168 (151, 178) | 0.61 |
| LDL-cholesterol (mg/dl) | Sitagliptin | 122 (110, 137) | 112 (102, 133) | 0.10 | 117 (108, 138) | 102 (80, 126) | 0.11 |
|  | Placebo | 130 (118, 138) | 111 (98, 137) | 0.26 | 118 (104, 123) | 103 (91, 110) | 0.69 |
| HDL-cholesterol (mg/dl) | Sitagliptin | 38 (32, 44) | 41 (40, 42) | 0.63 | 42 (41, 47) | 40 (40, 47) | 0.93 |
|  | Placebo | 40 (36, 44) | 41 (38, 45) | 0.97 | 42.5 (41.75, 43.25) | 42 (40, 42) | 0.17 |
| Triglyceride (mg/dl) | Sitagliptin | 151 (115, 213) | 166 (122, 214) | 0.24 | 123 (82, 163) | 123 (112, 140) | 0.41 |
|  | Placebo | 166 (138, 242) | 140 (104, 226) | 0.90 | 96 (87, 112) | 114 (102, 138) | 0.47 |
| ALT(IU/L) | Sitagliptin | 46 (36, 74) | 41 (27, 52) | 0.036 | 36 (26, 56) | 32 (22, 45) | 0.27 |
|  | Placebo | 44 (35, 58) | 40 (33, 50) | 0.078 | 29 (22, 38) | 30 (22, 32) | 0.80 |
| AST(IU/L) | Sitagliptin | 36 (29, 45) | 25 (20, 34) | <0.001 | 31 (26, 52) | 21 (16, 43) | 0.24 |
|  | Placebo | 32 (28, 41) | 26 (22, 30) | 0.013 | 26 (23, 39) | 26 (17, 34) | 0.61 |
| GGT(IU/L) | Sitagliptin | 48 (35, 55) | 41 (39, 43) | 0.13 | 42 (38, 54) | 44 (41, 50) | 0.41 |
|  | Placebo | 37 (24, 51) | 42 (31, 50) | 0.59 | 42 (36, 52) | 40 (40, 42) | 0.83 |
| ALKP(IU/L) | Sitagliptin | 195 (150, 216) | 177 (159, 228) | 0.77 | 225 (167, 254) | 195 (149, 234) | 0.17 |
|  | Placebo | 180 (165, 222) | 178 (168, 196) | 0.50 | 164 (154, 186) | 180 (168, 234) | 0.69 |
| Ferritin (µg/l) | Sitagliptin | 132 (69, 182) | 106 (61, 181) | 0.52 | 52 (26, 88) | 93 (67, 131) | 0.010 |
|  | Placebo | 118 (66, 228) | 67 (54, 134) | 0.26 | 30 (12, 85) | 39 (19, 78) | 0.55 |
| Fibrosis score, Med (IQR) | Sitagliptin | 6.20 (6.00, 7.30) | 5.70 (5.18, 6.23) | <0.001 | 6.8 (5.95, 7.25) | 6.7 (5.45, 7.70) | 0.54 |
|  | Placebo | 5.60 (4.95, 6.35) | 5.40 (4.80, 6.22) | 0.38 | 6.7 (5.97, 7.25) | 5.75 (4.92, 7.18) | 0.31 |

^a^ Median (IQR)

^b^ Wilcoxon signed rank test with continuity correction; Wilcoxon signed rank exact test

Abbreviation: FBS, fasting blood sugar; HOMA-IR: homeostasis model of insulin resistance; LDL: low-density lipoprotein; HDL: high-density lipoprotein; ALT: alanine aminotransferase; AST: aspartate aminotransferase; GGT: gamma-glutamyltransferase.

**Supplementary Table 3. Changes in outcome in the treatment and placebo groups based on the BMI status by sex ^a^**

|  |  |  | **Male (n=57 )** | | |  | **Female (n=19)** | | |
| --- | --- | --- | --- | --- | --- | --- | --- | --- | --- |
| **Variables** | **Groups** | **BMI status** | **Before intervention,**  **Median (IQR)** | **After intervention,**  **Median (IQR)** | **P-value ^b^** |  | **Before intervention,**  **Median (IQR)** | **After intervention,**  **Median (IQR)** | **P-value ^b^** |
| **ALT(IU/L)** | Sitagliptin | Normal | 75 (72, 75) | 46 (44, 49) | 0.50 |  | 35(32, 46) | 26(22, 58) | >0.99 |
|  |  | Overweight | 40 (33, 51) | 38 (22, 50) | 0.22 |  | 63(63.0, 63.0) | 49(49.0, 49.0) | >0.99 |
|  |  | Obese | 55 (38, 74) | 50 (38, 66) | 0.44 |  | 36(25, 47) | 32(22, 36) | 0.30 |
|  | Placebo | Normal | 56 (50, 61) | 42 (38, 46) | 0.25 |  |  |  |  |
|  |  | Overweight | 42 (35, 73) | 36 (31, 50) | 0.28 |  | 21(18, 45) | 31(30, 32) | >0.99 |
|  |  | Obese | 37 (34, 49) | 48 (40, 53) | 0.75 |  | 29(29, 31) | 22(21, 73) | 0.85 |
| **AST(IU/L)** | Sitagliptin | Normal | 65 (50, 66) | 41 (36, 46) | 0.50 |  | 26(24, 38) | 21(18, 41) | >0.99 |
|  |  | Overweight | 32 (28, 40) | 24 (20, 30) | 0.039 |  | 76(76, 76) | 65(65, 65) | >0.99 |
|  |  | Obese | 41 (31, 43) | 27 (18, 34) | 0.009 |  | 31(28, 43) | 20(15, 24) | 0.35 |
|  | Placebo | Normal | 42(37.5, 43.0) | 29(28.0, 36.5) | 0.50 |  |  |  |  |
|  |  | Overweight | 31 (28, 46) | 23 (19, 29) | 0.037 |  | 25(24, 37) | 31(28, 34) | >0.99 |
|  |  | Obese | 28.5 (26.0, 31.8) | 24(23.0, 28.0) | 0.75 |  | 27(23, 38) | 17(16, 29) | 0.62 |
| **GGT(IU/L)** | Sitagliptin | Normal | 67 (58, 82) | 53 (48, 58) | 0.50 |  | 60(58, 62) | 51(46, 80) | >0.99 |
|  |  | Overweight | 42 (35, 52) | 41 (36, 43) | 0.48 |  | 40(40, 40) | 48(48, 48) | >0.99 |
|  |  | Obese | 44 (38, 63) | 41 (39, 42) | 0.44 |  | 41(34, 42) | 43(41, 47) | 0.20 |
|  | Placebo | Normal | 46(43.50, 48.50) | 48 (45.00, 49.00) | >0.99 |  |  |  |  |
|  |  | Overweight | 34 (21, 42) | 38 (30, 49) | 0.41 |  | 51(33, 53) | 40(29, 40) | 0.50 |
|  |  | Obese | 44 (25, 53) | 41 (34, 46) | >0.99 |  | 41(40, 44) | 41(40, 137) | >0.99 |
| **ALKP(IU/L)** | Sitagliptin | Normal | 163 (149, 166) | 134 (128, 140) | 0.50 |  | 278(262, 416) | 195(180, 206) | 0.25 |
|  |  | Overweight | 195 (150, 204) | 172 (159, 194) | 0.43 |  | 253(253, 253) | 249(249, 249) | >0.99 |
|  |  | Obese | 212 (190, 238) | 228 (198, 256) | 0.074 |  | 174(151, 220) | 192(129, 234) | 0.94 |
|  | Placebo | Normal | 176 (139, 180) | 195 (151, 196) | 0.25 |  |  |  |  |
|  |  | Overweight | 170 (164, 216) | 176 (168, 195) | 0.35 |  | 162(159, 176) | 180(166, 211) | >0.99 |
|  |  | Obese | 227 (180, 270) | 176 (175, 180) | 0.50 |  | 165(150, 184) | 202(172, 244) | 0.62 |
| **Fibro score** | Sitagliptin | Normal | 7.90 (7.10, 12.20) | 5.10 (5.00, 8.10) | 0.25 |  | 6(5.75, 7.50) | 5.50(5.45, 7.00) | 0.17 |
|  |  | Overweight | 6.10 (5.80, 7.00) | 5.40 (5.00, 6.20) | 0.032 |  | 10.30 (10.30, 10.30) | 8.30 (8.30, 8.30) | >0.99 |
|  |  | Obese | 6.30 (6.00, 7.30) | 6.00 (5.70, 6.10) | 0.068 |  | 6.80(5.95, 7.00) | 6.70(6.00, 7.05) | 0.67 |
|  | Placebo | Normal | 4.60 (4.60, 6.30) | 6.60 (5.30, 6.70) | >0.99 |  |  |  |  |
|  |  | Overweight | 5.50 (5.00, 6.07) | 5.10 (4.75, 6.00) | 0.43 |  | 5(4.85, 6.50) | 6(4.95, 7.40) | 0.50 |
|  |  | Obese | 6.55 (5.72, 7.15) | 5.65 (5.25, 6.43) | 0.88 |  | 6.90(6.50, 7.00) | 5.50(5.00, 7.00) | 0.12 |

^a^ Median (IQR), ^b^ Wilcoxon signed rank test with continuity correction; Wilcoxon signed rank exact test
